# Comparative Utility of Acupuncture and Western Medication in the Management of Perimenopausal Insomnia: A Systematic Review and Meta-Analysis

**DOI:** 10.1155/2021/5566742

**Published:** 2021-04-26

**Authors:** Fei-Yi Zhao, Qiang-Qiang Fu, Gerard A. Kennedy, Russell Conduit, Wen-Zhong Wu, Wen-Jing Zhang, Zhen Zheng

**Affiliations:** ^1^School of Health and Biomedical Sciences, RMIT University, Bundoora, Victoria 3083, Australia; ^2^Shanghai Municipal Hospital of Traditional Chinese Medicine, Shanghai University of Traditional Chinese Medicine, Shanghai 200071, China; ^3^Department of Nursing, School of International Medical Technology, Shanghai Sanda University, Shanghai 201209, China; ^4^Yangpu Hospital, Tongji University School of Medicine, Shanghai, China; ^5^School of Science, Psychology and Sport, Federation University, Mount Helen, Victoria, Australia; ^6^Institute for Breathing and Sleep, Austin Health, Heidelberg, Victoria 3084, Australia; ^7^Jiangsu Province Hospital of Chinese Medicine, Affiliated Hospital of Nanjing University of Chinese Medicine, Nanjing, Jiangsu, China

## Abstract

**Background:**

Many women with perimenopausal insomnia (PMI) have sought alternative therapies such as acupuncture because of concerns about risks associated with hormone replacement therapy (HRT) and/or psychotropic drugs. This systematic review aimed to clarify if acupuncture alone or combined with standard Western pharmacotherapy (HRT and/or psychotropic drugs) is more effective in ameliorating PMI in comparison to pharmacotherapy alone.

**Methods:**

Randomized controlled trials (RCTs) of PMI treatment via acupuncture alone or combined with Western pharmacotherapy versus Western pharmacotherapy were searched for from eleven databases from inception to March 2020. Cochrane criteria were followed.

**Results:**

Fifteen studies involving 1410 women were analyzed. Meta-analysis indicated that acupuncture significantly reduced the global scores of Pittsburgh Sleep Quality Index (PSQI) [MD = −2.38, 95% CI (−3.38, −1.37), *p* < 0.01] and Kupperman Index [MD = −5.95, 95% CI (−10.68, −1.21), *p* = 0.01], compared with hypnotics. Acupuncture combined with hypnotics was more effective than hypnotics alone in decreasing PSQI scores [MD = −3.13, 95% CI (−5.43, −0.83), *p* < 0.01]. Too few RCTs were available to investigate the clinical efficacy differences between acupuncture and HRT/psychotropic drugs other than hypnotics.

**Conclusions:**

Despite limited evidence, in comparison to hypnotics, acupuncture was associated with significant improvements in PMI, and reductions of other menopausal symptoms. This finding suggests that acupuncture may be a useful addition to treatment for PMI.

## 1. Background

Perimenopausal insomnia (PMI) is characterized by difficulties with initiation and maintenance of sleep, and/or early morning awakening [1–4], but tends to stabilize as women transit to postmenopause [1, 5]. Around 59% of American women in midlife report that they experienced PMI symptoms at least a few nights weekly [6, 7]. The prevalence of PMI is higher in China and India, reaching at 65.86% [8] and 67% [9], respectively. Insomnia has many negative effects on physiological and psychological functioning. Insomnia leads to daytime fatigue, mental distress, decreased life quality, elevated risk of accident, and exacerbation of comorbid health conditions and predicts cognitive decline, substance abuse/dependence, and suicide [10–14].

Perimenopausal syndrome including PMI symptoms are often managed with hormone replacement therapy (HRT) [15–17]. Despite its benefits for sleep [16], HRT, particularly long-term use, is associated with increased risk of breast cancer, ovarian cancer, and cardiovascular diseases [16, 18–20]. Sedatives and hypnotics are also effective for insomnia [14, 21–23] but have limitations because of problems with tolerance, dependency and withdrawal, residual daytime sedation, risk of rebound insomnia, memory and cognitive impairments, and motor incoordination resulting in falls in the elderly [14, 22, 24, 25]. Evidence also supports the positive effect of behavioral and psychological therapy, particularly cognitive behavioral therapy (CBT) for menopause-related insomnia [26–28]. However, CBT is not widely available [24], and is expensive [24] and time consuming to administer [25]. Many women thereby seek complementary and alternative medicines (CAM) such as dietary therapy, herbal medicine, massage therapy, and acupuncture for symptomatic relief [29, 30].

Acupuncture is one of the most popular and safest CAM therapies [25] as part of ancient Traditional Chinese Medicine (TCM) with a history of more than 4000 years [31]. It is a traditional healing technique involving the insertion of fine, solid, metallic needles into targeted sites called “acupoints” on the body wall to achieve therapeutic outcomes [31–33]. After insertion, the needles are usually stimulated manually with slight twisting back and forth and with gentle movements up and down (manual acupuncture, MA), or are stimulated by the sequential electrical impulses delivered by an electric microcurrent device (electroacupuncture, EA) [31, 33, 34].

Several clinical trials regarding the use acupuncture for the treatment of PMI symptomatology have been published. However, the inclusion of trials with low quality of evidence and/or inconsistent of outcomes between the trials contributes challenges to drawing a definitive conclusion on the advantages and benefits of acupuncture [35–39]. Compared with HRT, or hypnotics/sedatives or other psychotropic drugs, how effective and safe is acupuncture? An unbiased estimate can allow more physicians to decide whether acupuncture is an alternative option. We carried out a systematic review and aimed to address the following research questions: (1) how effective and safe is acupuncture for the management of PMI in comparison with standard Western pharmacotherapy including HRT (e.g., nilestriol, tibilone, estradiol valerate, etc.), and psychotropic substances (e.g., hypnotic, sedative or other psychotropic drugs, etc.); (2) when acupuncture is used as an adjuvant therapy to standard Western pharmacotherapy, could it further enhance the therapeutic effect or reduce the side effects of the standard Western pharmacotherapy? Our systematic review was performed in accordance with Preferred Reporting Items for Systematic Reviews and Meta-Analysis (PRISMA) statement guidelines.

## 2. Materials and Methods

The protocol for this systematic review was registered in the Prospective Register of Systematic Reviews (PROSPERO): No. CRD42020170616.

### 2.1. Eligibility Criteria

Studies included were published randomized controlled trials (RCTs) with parallel designs. Women in the perimenopausal period with a clinical diagnosis of primary insomnia as per standard diagnostic criteria were included. Any trial without a standard diagnostic guideline was excluded even if it mentioned that the patient was diagnosed with PMI or it provided brief information regarding women's complaints of sleep disorders. Participants in a pre- or postmenopausal status, or with comorbid psychiatric disorders, other sleep complaints, and/or other diseases, were excluded. Intervention were restricted to traditional needle acupuncture (TNA) including MA and EA, or TNA combined with standard pharmacotherapy for PMI (HRT or psychotropic substances). Comparator interventions were restricted to standard Western pharmacotherapy for PMI. The primary outcome was self-reported, validated sleep scales (e.g., Pittsburgh Sleep Quality Index (PSQI), Insomnia Severity Index (ISI), Athens Insomnia Scale (AIS), etc.). Secondary outcomes included objective sleep parameters measured by sleep monitoring devices, perimenopausal symptoms assessed with validated scales (e.g., Kupperman Index (KI), Menopause-Specific Quality of Life (MENQOL), etc.), anxiety/depression symptoms, serum hormone levels (e.g., FSH, E2, LH, etc.), clinical effectiveness rate, and adverse events.

### 2.2. Search Strategy and Data Extraction

Four Chinese and seven English electronic databases—China National Knowledge Infrastructure (CNKI), Chongqing VIP database (CQVIP), Wanfang database, China biomedical literature service system (SinoMed), and Cochrane Central Register of Controlled Trials (CENTRAL), Sciverse ScienceDirect, MEDLINE (via PubMed), EMBASE, Springer, Allied and Complementary Medicine Database (AMED), and PsycINFO (ProQuest)—with language restrictions of Chinese and English, were searched from the inception date of each database until March 2020. Additional studies were also identified from other sources, including the online trial registries such as US ClinicalTrials.gov, and WHO International clinical trials registry platform search portal, the reference lists of the included papers, existing systematic reviews, and grey literatures (Appendix 1).

EndNote software (Version X7) was used to store the results of search and to remove duplicate articles (if multiple literature reports were judged to be the same trial, the one with the largest sample size and the most comprehensive information was retained). One researcher (QQF) firstly generated the search strategy, searched the potential databases, and drew up a list of all the records. Two evaluators (FYZ and QQF) independently assessed and screened the articles according to the inclusion and exclusion criteria. Any inconsistency and/or disagreement was settled by consensus or arbitration by a third reviewer (ZZ). Finally, two reviewers (FYZ and QQF) independently extracted the data and proofread the information.

For each study, the following data for demographic and clinical characteristics were extracted: the last name of the first author, publication year, grouping methods and number of patients in each group, duration of insomnia, diagnostic criteria used, TCM syndrome type of patients, protocols including timing, frequency and dosage in acupuncture, the acupoints selected, prescription in control group (type, dosage, and oral frequency of Western medication), outcome measures, results, follow-up, and adverse events. Incomplete data or queries were followed up with the corresponding authors of the original papers via emails.

### 2.3. Study Quality and Risk of Bias Assessment

We used Cochrane Collaboration's risk of bias tool to deter risk of bias and assess the internal validity among included RCTs [40]. The methodological quality of each RCT was appraised against seven specific domains: (1) random sequence generation; (2) allocation concealment; (3) blinding of participants and personnel; (4) blinding of outcome assessment; (5) incomplete outcome data; (6) selective reporting; (7) other bias, which was evaluated in light of baseline balance, and source of funding or conflict of interest. A bias value of “high,” “unclear,” or “low” was appraised and assigned to each domain [40]. The revised Standards for Reporting Interventions in Clinical Trials of Acupuncture (STRICTA) checklist (revised version, published on year 2010) was used to evaluate and describe the details of acupuncture procedure including completeness and reporting quality in each RCT [41]. The items covered by STRICTA involved (1) acupuncture rationale, (2) needling details, (3) treatment regimen, (4) other components of treatment, (5) practitioner background, and (6) control or comparator interventions.

## 3. Data Analysis

The meta-analysis was performed via Cochrane Collaboration Review Manager Software (RevMan Version 5.3). Given that the major outcome measures (global scores of scales) were continuous variables, mean differences (MD) were analyzed. When serum hormone levels were presented in the different units of measurement, standardized mean differences (SMD) were used. Confidence intervals (CIs) were established at 95%. Dichotomous data such as clinical effectiveness rate were reported as risk ratio (RR) with 95% CI. Level of heterogeneity across studies was tested using the *Q*-test and *I*^*2*^ test. Statistical significance was set at two-tailed probability (*p*) value <0.05. The results were pooled using a fixed effects model when the *p* value was >0.10 in the *Q*-test and the *I*^*2*^ value was ≤50% which was considered to an acceptable level of heterogeneity. Otherwise, a random effects model was applied. When significant heterogeneity existed, subgroup analysis was carried out based on different acupuncture stimulations (MA or EA), different prescriptions in the controls (different kinds of HRT/psychotropic drug used), or different acupuncture methods (body acupuncture alone or body acupuncture combined with scalp acupuncture). Sensitivity analysis and meta-regression analysis were also adopted to explore sources of heterogeneity and check robustness of the conclusions. Publication bias was investigated via Egger's test and Begg's test.

## 4. Results Analysis

**Figure 1:**
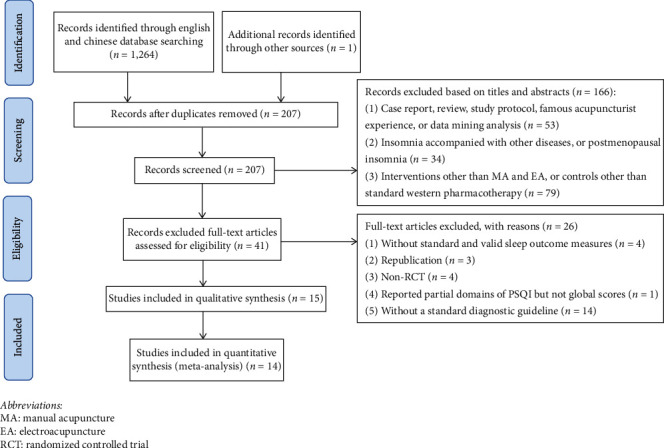
Flow diagram of the study selection process. MA, manual acupuncture; EA, electroacupuncture; RCT, randomized controlled trial.

The initial search yielded 1265 potentially eligible studies. After removing the duplicates, we screened titles and abstracts of 207 remaining records, and 166 records were excluded. Eventually, 15 studies met the predefined criteria (Figure 1). All included studies were qualitatively analyzed, and 14 of them underwent quantitative synthesis (meta-analysis).

### 4.1. Description of Studies

Four out of the 15 RCTs [42–46] investigated the clinical efficacy of EA, while the remaining 11 RCTs investigated the effects of MA. Acupuncture treatment was provided daily up to three times per week from 20 days up to three months. In the 14 studies using psychotropic drugs, only common hypnotics, such as Estazolam (11/14 trials), Alprazolam (2/14) and Eszopiclone (1/14), were used. In the only RCT [42] comparing acupuncture with HRT, progynova combined with medroxyprogesterone acetate was employed as a control. We did not identify any eligible papers for comparisons between HRT and acupuncture combined with HRT (Table 1).

Table 2 and Appendix 2 show the assessment time-points and results of each outcome in each trial. PSQI was used in fourteen RCTs [42, 44–56] and AIS was used in the remaining one RCT [43] to assess changes in sleep at pre- and posttreatment. In addition, KI [42, 44, 47, 48, 53] and serum FSH [42, 44, 50, 52, 55], E2 [42, 44, 50, 52, 55], and LH [50, 52] were employed to evaluate patients' perimenopausal symptoms.

Amongst the included trials, Chinese Classification of Mental Disorders, 3rd Edition (CCMD-3), was most frequently used for the diagnosis of insomnia (nine studies, 60.00%) [44, 47–49, 50, 51, 54–56] followed by Criteria of Diagnosis and Therapeutic Effect of Diseases and Syndromes in TCM (CDTE-TCM) (four studies, 26.67%) [43, 48, 50, 53], Chinese Classification of Mental Disorders, 2nd Edition (CCMD-2) (three studies, 20.00%) [42, 46, 52], International Classification of Diseases, 10th edition (ICD-10) (two studies, 13.33%) [50, 51], Diagnostic and Statistical Manual of Mental Disorders, 4th Edition (DSM-IV) (one study, 6.67%) [43], and Guidelines for Diagnosis and Treatment of Insomnia in Chinese Adults, 2012 Edition (GDTICA) (one study, 6.67%) [53]. It could be noticed that four trials [43, 47, 50, 51] set strict diagnostic criteria; that is, only those insomniacs who met both the criteria of certain TCM pattern and the diagnostic criteria of Western medicine were included. Such a research design that adopts the dual diagnostic criteria of Chinese and Western medicine is worth recommending since it is more in line with the research norms of TCM or “Integrated Medicine.”

Seven studies reported adverse events (AEs) [42–44, 47, 48, 51, 53]. AEs associated with acupuncture treatment included hematoma (8/136) [42, 48, 51, 53], mild dizziness (1/37) [42], and mild tension (6/41) [44]; AEs associated with HRT included breast tenderness (2/36) [42], mild headache (1/36) [42], and colporrhagia (1/36) [42]; AEs associated with hypnotics included dizziness (31/137) [44, 48, 51, 53], daytime sleepiness and fatigue (31/107) [44, 51, 53], mild nausea (1/33) [47], thirst (2/33) [48], memory loss (2/30) [53], and development of drug dependence (8/32) [43] (Table 1).

### 4.2. Study Quality Evaluation

#### 4.2.1. Risk of Bias Assessment

**Figure 2:**
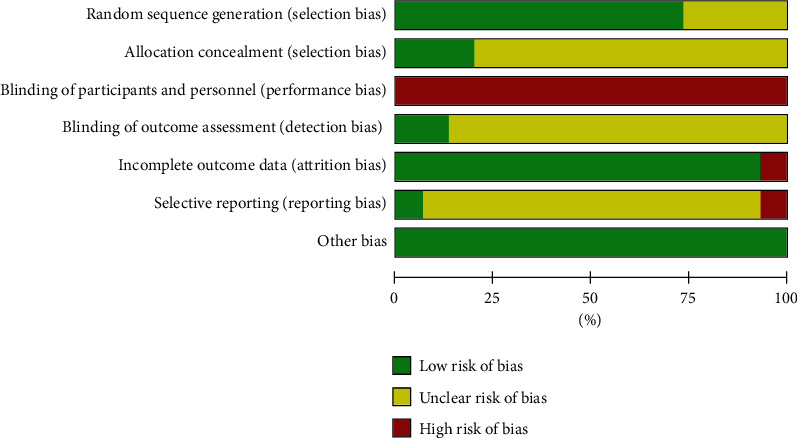
Risk of bias summary. Other biases are assessed based on baseline balance and source of funding or conflict of interest.

Eleven out of 15 trials provided an adequate description of the process and method of randomization [42, 44–48, 50–51, 53, 55, 56], while four trials [43, 49, 52, 54] only mentioned that the RCT design was employed in the trial but did not clarify the specific randomization procedure. Twelve trials were judged as being unclear in risk of bias in the domain of allocation concealment [42–44, 47, 49–50, 52–56]. Only two trials [48, 51] reported blinding of outcome assessment. For the item of selective outcome reporting, one RCT [51] was assessed as low risk of bias as it was registered in the ChiCTR with a protocol, and one RCT [50] was assessed as high risk of bias as it mentioned a 30-day follow-up plan in the methods section but did not report any valid follow-up data. The remaining 13 studies were rated as unclear in risk of bias because protocols were not always available or there was insufficient evidence and information to permit a clear judgment. All 15 studies addressed baseline balance adequately. However, only five studies [42, 51–53, 56] explicitly reported the financial supports and declared no competing financial interests and were judged at low risk of bias in this domain (Figure 2, Appendix 3 and Appendix 4).

#### 4.2.2. Study Completeness and Reporting Quality Assessment

Traditional Chinese acupuncture was used in all the included studies, and all the acupuncture treatment was provided in accordance with the TCM theory. All the 15 trials reported the needle stimulation (MA or EA), name and selection rationale of the acupoints used, and response sought described as *De-qi*. All except for one trial [55] gave the information of the needle retention time ranging from 20 to 40 minutes. The depth of insertion was only clearly shown in 11 trials [42–46, 48–52, 54]. The needle type was presented in detail in only ten studies [42, 43, 46–48, 50–51, 54, 56]. As the core part of acupuncture therapy, the intervention regimen including treatment frequency, dosage, and duration was clearly and completely reported in all the studies. Setting of treatment and acupuncturist background were not illustrated in any included trial. All other items in each RCT were reported completely (Appendix 5).

### 4.3. Analysis of Outcome Measures

The qualitative and quantitative analysis for outcome measures in the 15 included studies were divided into three parts: (1) acupuncture versus HRT (*n* = 1); (2) acupuncture versus hypnotics (*n* = 11); (3) acupuncture combined with hypnotics versus hypnotics (*n* = 3) (Appendix 6).

#### 4.3.1. Acupuncture vs HRT

Only one study [42] compared acupuncture (intervention: EA) with HRT (control: progynova + medroxyprogesterone acetate). Both therapies significantly decreased PSQI, KI, and MENQOL scores, and increased E2 levels. Compared with HRT, EA was more effective in reducing PSQI scores but less effective in downregulating FSH levels and upregulating E2 levels. There was no statistical group difference in clinical effectiveness rate, or in reducing KI and MENQOL scores. At the 3-month follow-up, all improvements maintained in both acupuncture and HRT groups with no group difference except for the scores of PSQI, which were lower in the acupuncture group (Table 2).

#### 4.3.2. Acupuncture vs Hypnotic

Eleven trials were included in this comparison [43–53]. Meta-analysis was performed for five indicators, including PSQI, KI, FSH, E2, and clinical effectiveness rate. We did not carry out meta-analysis for other outcome measures because there were fewer than three included studies for each of them (Appendix 2).

**Figure 3:**
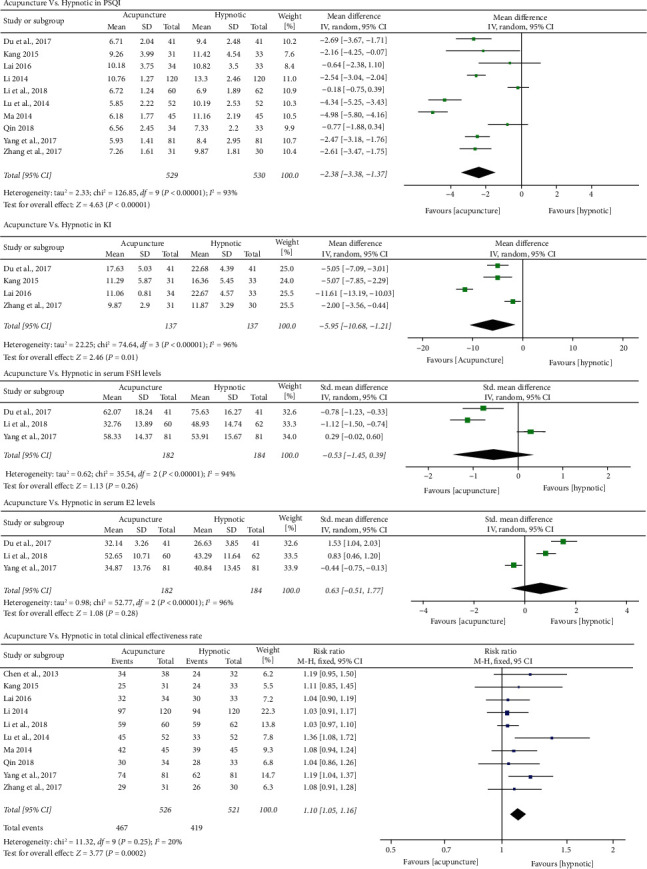
Forest plots of acupuncture vs hypnotic in PSQI, KI, serum FSH and E2 levels, and total clinical effectiveness rate.

*(1) Insomnia Symptoms.* Ten [44–53] out of 11 trials employed PSQI as an outcome measure. Due to the high heterogeneity (*p* < 0.01, *I*^*2*^ = 93%), a random effects model was used. The results favored acupuncture in reducing PSQI global scores [MD = −2.38, 95% CI (−3.38, −1.37), *p* < 0.01] (Figure 3). Another study [43] used AIS as the outcome. Considering the potential impact, we also included it and analyzed the pooled estimate effects of all 11 studies based on SMD. However, the results did not significantly change and still favored acupuncture in alleviating insomnia symptoms [SMD = −1.05, 95% CI (−1.44, −0.65), *p* < 0.01] (Appendix 7).

Ten [43, 46–53] studies assessed the clinical effectiveness rates of both acupuncture and hypnotics for PMI (Appendix 8). The meta-analysis results favored acupuncture for the total effectiveness rate for PMI [RR = 1.10, 95% CI (1.05, 1.16), *p* < 0.01] (Figure 3).

*(2) Perimenopausal Symptoms and Hormonal Regulation.* Four trials [44, 46–47, 53] employed KI as an outcome measure. The results favored acupuncture in reducing KI scores [MD = −5.95, 95% CI (−10.68, −1.21), *p* = 0.01] (Figure 3).

Three trials [44, 50, 52] also reported FSH and E2 levels, and all supported acupuncture significantly downregulating FSH and upregulating E2 levels. However, no significant differences were identified between acupuncture and hypnotics in regulating either FSH [SMD = −0.53, 95% CI (−1.45, 0.39), *p* = 0.26] or E2 levels [SMD = 0.63, 95% CI (−0.51, 1.77), *p* = 0.28] (Figure 3).

(3) *Subgroup Analysis.* We found a significant interaction effect between different type of hypnotics (Estazolam vs Alprazolam vs Eszopiclone, Chi^2^ statistic 27.34, df = 1, *p* < 0.01) on PSQI, suggesting acupuncture was better than Estazolam, but not when compared with Alprazolam or Eszopiclone, in reducing PSQI scores; however, there was only one study each for the latter two drugs. We also found interaction effect between different type of hypnotics (Estazolam vs Eszopiclone, Chi^2^ statistic 30.43, df = 1, *p* < 0.01) on KI; similarly, only one study included the Eszopiclone subgroup. No interaction effects were identified in other subgroups (Appendix 9).

*(4) Sensitivity Analysis*. In an attempt to address the high heterogeneity, sensitivity analysis was performed based on the outcome of PSQI to ensure the results were not due to one or two studies. We chose influence analysis, by removing one study at a time and recalculating the combined estimate on the remaining studies to evaluate the stability of the results. We did not perform sensitivity analysis for the other outcome measures because of the small number of studies (<10).

**Figure 4:**
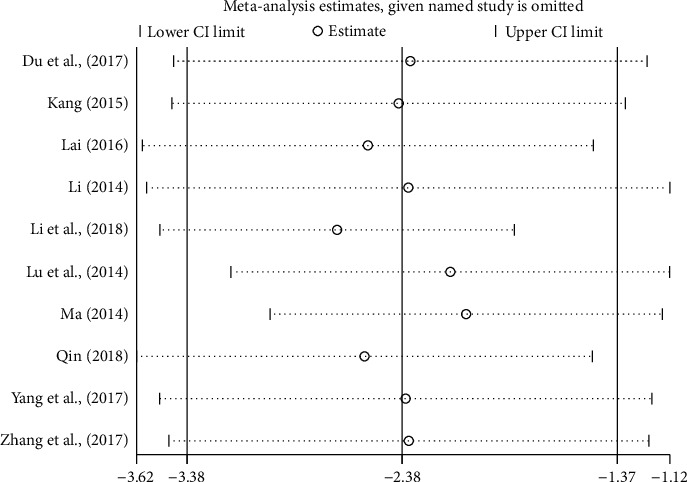
Sensitivity analysis based on PSQI.

The results indicated that each single study had little impact on the pooled estimate effects of PSQI, and the overall robustness and reliability of our study results was relatively high (Figure 4).

*(5) Meta-Regression Analysis.* Using PSQI as the outcome measure, we conducted univariate meta-regressions to investigate the sources of heterogeneity by treating study sample size, acupuncture stimulation, and acupuncture methods as covariates and conducted multifactor meta-regressions by treating types of hypnotics as covariates. However, the heterogeneity across the 10 included studies could not be substantially explained by study sample size (*I*^*2*^ = 93.69%, Tau2 = 2.49, *p* = 0.86), acupuncture stimulation (*I*^*2*^ = 91.34%, Tau2 = 1.79, *p* = 0.14), acupuncture methods (*I*^*2*^ = 92.13%, Tau2 = 2.01, *p* = 0.24), and types of hypnotics (*I*^*2*^ = 86.70%, Tau2 = 1.46, *p* = 0.13) (Appendix 10, Supplemental Figures 1–3).

#### 4.3.3. Acupuncture Combined with Hypnotic vs Hypnotic

Three trials [54–56] were included. Meta-analysis was only carried out for PSQI but not for other outcomes because there were fewer than three included trials for each of them.

**Figure 5:**
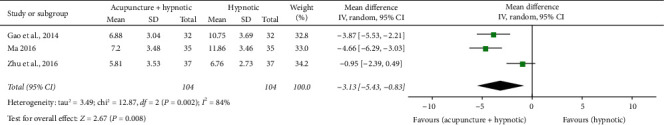
Forest plot of acupuncture + hypnotic vs hypnotic in PSQI.

*(1) Insomnia Symptoms.* PSQI was employed as an outcome in all three trials which compared MA combined with Estazolam to Estazolam alone. The results favored MA combined with Estazolam [MD = −3.13, 95% CI (−5.43, −0.83), *p* < 0.01] (Figure 5).

*(2) Perimenopausal Symptoms and Hormonal Regulation.* No study reported perimenopausal symptoms. One trial [55] reported the outcomes of hormones levels. Both therapies significantly downregulated FSH levels and upregulated E2 levels with results favoring MA combined with Estazolam.

### 4.4. Publication Bias Test

**Figure 6:**
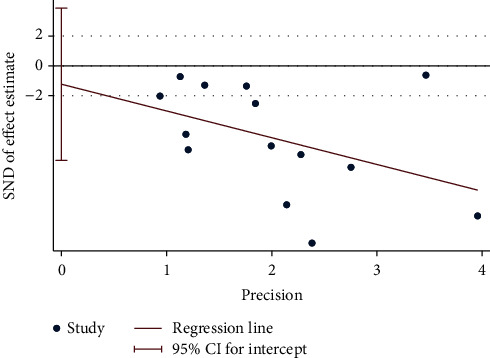
Publication bias test based on PSQI.

We used linear regression analysis (Egger's test) to detect the publication bias based on PSQI. According to the funnel plots, linear regression analysis obtained *p* = 0.12 (Figure 6), suggesting no significant publication bias was identified in PSQI. We did not conduct a publication bias test for the other outcome measures because of the small number of studies (<10).

## 5. Discussion

### 5.1. Summary of Findings

Acupuncture alone or combined with hypnotic drugs is superior to hypnotics drugs alone in improving sleep quality and quantity in perimenopausal women. The reduction of PSQI global score varied from 2.4 to 3.1 points, reflecting the clinical significance. Whether or not the results of acupuncture were mediated via regulating serum hormone levels, such as FSH and E2, remains unclear because there was insufficient data. Differences in the effect of acupuncture in comparison to HRT are also not clear because there was only one study with a small sample that addressed this comparison. No studies reported if acupuncture could reduce the side effects of HRT or hypnotic drugs. Acupuncture appeared to be well-tolerated and safe as the adverse events were few and only mild. The most frequent adverse event was hematoma, which usually healed quickly after the needles were removed. Overall, the quality of the studies was low to moderate due to a lack of blinding of patients and outcome assessors.

### 5.2. Strengths, Limitations, and Comparison with Previous Systematic Reviews

A previous systematic review has confirmed the effect of acupuncture over sham acupuncture in improving PMI [57]. To the best of our knowledge, this was the first systematic review and meta-analysis investigating the effectiveness and safety of acupuncture versus standard Western pharmacotherapy in improving PMI. Women in Western countries are not likely to immediately give up Western medicine and choose acupuncture. However, they may be more willing to adopt acupuncture as adjuvant therapy to Western medication as part of a comprehensive management program [58, 59]. Our review specifically addresses this question and supports a better effect of acupuncture alone or when combined with hypnotics against hypnotics alone.

Previous systematic reviews included many different forms of acupoint-based therapies, such as scrape therapy [38], moxibustion [36–38, 57], acupressure [37, 57], point application [60], and acupoint catgut implantation [38, 39]. Such practice introduces extra variability and makes it difficult to understand the effect of acupuncture. We only focused on common forms of acupuncture (i.e., MA or EA) to reduce variability and to better reflect the real clinical practice. We also aimed to understand the potential factors mediating the hypnotic effect of acupuncture in perimenopausal women by analyzing data about perimenopausal symptoms and hormonal levels, which was not included in previous reviews.

Several limitations in this review should be acknowledged. First, the meta-analysis was limited by the number of studies and small sample sizes despite our comprehensive search. Second, the quality of included studies was less than satisfactory based on the Cochrane Collaboration's risk of bias tool. Third, some deficiencies in the reporting quality of included RCTs are additional reasons for lowering the evidence quality. For instance, among the 15 RCTs, only two trials clearly provided the methods for sample size estimation [48, 51], two trials included follow-ups for assessing the mid- or long-term effects of the study interventions [42, 50], and four trials reported review process of the human research ethics committee [44, 46, 51, 55]. Fourth, the heterogeneity was high across the studies. We employed subgroup, sensitivity, and meta-regression analysis but could not identify the sources. It was likely to have been contributed to by variations in treatment dosage and frequency, acupoints selections/combinations, and/or electrical stimulation parameters in those EA-related trials between studies. Fifth, all the included psychotropic drugs are hypnotics, so it remains unclear if acupuncture is more effective and safer than other drugs such as Mirtazapine (antidepressant) [61] and Quetiapine (antipsychotic drug) [62] that are also widely used for insomnia in the clinical practice. Finally, all the included RCTs were conducted in China. It is unknown if the results could be replicated in women beyond China. Further rigorous and well-designed RCTs with larger sample sizes, effective follow-ups, and multi-center design were required to build stronger evidence.

Considering the consistency in findings, and deficiency in study quality, we rate the strength of evidence being low to moderate, supporting the positive effect of acupuncture.

### 5.3. Interpretation of Findings

Our review found acupuncture was better than hypnotic drugs alone in reducing PSQI score by 2.4–3.1, which is clinically significant. One study [42] also demonstrated the long-term effect of acupuncture. That is, three months' acupuncture (three sessions weekly) demonstrated improvement in sleep maintained for at least three months. Frequent relapse is typical of insomnia [21], and it is one of the major reasons for numerous insomniacs reject sedative or other psychotropic drugs [21, 22]. The potential long-term effect of acupuncture warrants investigations in future trials.

It is interesting to note that acupuncture also improved perimenopausal symptoms (decreased KI scores), better than hypnotic drugs, reflecting different underlying mechanisms of the two interventions. There is some evidence supporting acupuncture increasing E2 and decreasing FSH. Previous studies showed that decreasing E2 is associated with higher odds of difficulty in initiating and staying asleep and increasing FSH is associated with higher odds of frequent nocturnal awakenings [63, 64]. Another preclinical study confirmed the modulatory effects of E2 replacement in spontaneous or sleep deprivation-induced c-Fos expression in sleep/wake-regulatory and limbic forebrain nuclei [65]. Therefore, it could be hypothesized that acupuncture improves PMI by modulating sex hormones. This is somewhat similar to the mechanism of HRT on PMI, as HRT is reported to regulate sleep through acting on the E2 and E2 receptors in the central nervous system [66]. A previous systematic review also showed a substantial association of acupuncture with improved sleep disturbances in women with PMI or postmenopausal insomnia [60]. Furthermore, that review demonstrated that the association of reduction in menopause-related sleep disturbance and acupuncture was correlated with increase in serum E2 levels [60]. However, in our review, the changes in those hormones in the acupuncture group did not differ from those in hypnotic drugs group, or those in HRT group as shown in the only one HRT study. Whether hormonal regulation mediates the effect of acupuncture on PMI requires further investigation.

In addition, KI measures both somatic and mental perimenopausal symptoms, including anxiety, depression, and vasomotor symptoms (e.g., hot flashes, sweating, etc.) [67]; all of these could contribute to sleep disturbance in perimenopausal women [63, 66]. Included studies in this review did not report which specific symptoms in KI were improved. Future studies also need to include specific scales/questionnaires for depression and anxiety.

The second aim of this systematic review was to investigate acupuncture as an adjuvant therapy to hypnotic drugs, whether acupuncture can further enhance the clinical efficacy and/or reduce the adverse reactions caused by these Western medications. While three RCTs [54–56] in this category showed the combined therapy was more significantly effective in improving PMI than hypnotics, none reported if acupuncture also reduces the side effects of those medications. Future studies are thereby needed to explore the safety of a combined therapy of acupuncture and hypnotics, as well as the therapeutic effects and safety of a combined therapy of acupuncture and HRT/other psychotropic drugs.

Given none of the studies included sham acupuncture, it is difficult to know if the positive effect of acupuncture was due to placebo effects as patients were not blinded and we were also not clear if the outcome assessors were blinded. To understand the placebo effects of acupuncture and underling mechanism, future studies need to include objective measures, such as PSG, as they are conducive to a clearer understanding of the effects of acupuncture on sleep physiology indicated by sleep architecture.

## 6. Conclusions

Low to moderate level of evidence supports that acupuncture could be a safe alternative to or adjuvant to hypnotic drugs in improving sleep quality and quantity as well as other menopause-related symptoms among women with PMI. Future studies need to clarify whether acupuncture could also be an adjuvant to HRT, as well as include sham acupuncture in the trial designs and utilize PSG to confirm the effect of acupuncture over common drugs for PMI and to understand if the clinical improvement is associated with sleep architecture changes induced by acupuncture.

## Figures and Tables

**Table 1:** Study characteristics of 15 included studies.

Author, year	Group/size	Age (year)	Insomnia duration (m = month, act y = year)	Diagnostic system	TCM syndrome type	Acupuncture interventions	Acupoints	Prescription in control group (Western medication)	Outcome measure tool	Acupuncture/acupuncture + Western medication compared with Western medication	Follow-up	Adverse events
Ma et al. 2017 [42]	(i) EA/*n* = 37 (ii) Progynova + medroxyprogesterone/*n* = 36	(i) EA/49.77 ± 2.68 (ii) Progynova + medroxyprogesterone acetate/49.11 ± 2.10	(i) EA/17.70 ± 9.93m (ii) Progynova + medroxyprogesterone acetate/18.27 ± 8.61 m	CCMD-2	NR	(i) 30 min/day, 3 days/week for 12 weeks (ii) sparse-dense wave, 2/15 Hz	CV4; EX-CA1; EX-HN3; HT7; SP6; ST25	(i) A total of 3 treatment cycles. Each treatment cycle includes Progynova 1 mg daily for 21 consecutive days (with medroxyprogesterone acetate 10 mg daily added from day 14 to day 21) and then stop medication for 7 days	(i) PSQI (ii) KI (iii) MENQOL (iv) FSH (v) E2	(i) Compared with Progynova + medroxyprogesterone acetate *p* < 0.05 (ii) Compared with Progynova + medroxyprogesterone acetate *p* > 0.05 (iii) Compared with Progynova + medroxyprogesterone acetate *p* > 0.05 (iv) Compared with Progynova + medroxyprogesterone acetate *p* > 0.05 (v) Compared with Progynova + medroxyprogesterone acetate *p* < 0.05	(i) Available data for 3 months follow-up	(i) EA/*n* = 3 (two for hematoma; one for mild dizziness) (ii) Progynova + medroxyprogesterone acetate/*n* = 4 (two for breast tenderness; one for mild headache; one for colporrhagia)
Chen et al. 2013 [43]	(i) EA/*n* = 38 (ii) Alprazolam/*n* = 32	(i) EA/48.00 ± 6.00 (ii) Alprazolam/48.00 ± 7.00	(i) EA/6.90 ± 0.20 m (ii) Alprazolam/6.50 ± 0.30 m	DSM-IV, CDTE-TCM	NR	(i) 30 min/day for 20 days (7 days off every 10 days) (ii) Continuous wave, 0.7 Hz	EX, GV20, HT7, KI3, KI7, KI10, LR3, PC6, SP6, SP9, SP10	(i) Alprazolam 0.4 mg/day for 20 days	(i) AIS	(i) Compared with Alprazolam *p* < 0.05	No follow-up	(i) EA/*n* = 0 (ii) Estazolam/*n* = 8 (development of drug dependence after treatment)
Du et al. 2017 [44]	(i) EA/*n* = 41 (ii) Estazolam/*n* = 41 (iii) Herbal medicine/*n* = 41 (iv) EA + Herbal medicine/*n* = 42	(i) EA/50.17 ± 2.46 (ii) Estazolam/50.45 ± 3.19 (iii) Herbal medicine/49.76 ± 3.05 (iv) EA + Herbal medicine/50.61 ± 2.62	(i) EA/2.33 ± 0.72 y (ii) Estazolam/2.06 ± 0.85 y (iii) Herbal medicine/1.93 ± 1.05 y (iv) EA + Herbal medicine/1.96 ± 0.99 y	CCMD-3	NR	(i) 30 min/day, 6 days/week for 4 weeks (ii) Continuous wave, >50 Hz	PC6, SP6, Sishenzhen (1.5 *Cun* apart from GV20), Dingshenzhen (0.5 *Cun* up to EX-HN3, and 0.5 *Cun* up to GB14)	(i) Estazolam 1 mg/day, 7 days/week for 4 weeks	(i) PSQI (ii) KI (iii) WHOQOL-BREF (iv) FSH (v) E2	(i) Compared with Estazolam *p* < 0.05 (ii) Compared with Estazolam *p* < 0.05 (iii) Compared with Estazolam *p* < 0.05 (iv) Compared with Estazolam *p* < 0.05 (v) Compared with Estazolam *p* < 0.05	No follow-up	(i) EA/*n* = 6 (mild tension before EA) (ii) Estazolam/*n* = 26 (dizziness, daytime sleepiness) (iii) Herbal medicine/*n* = 4 (gastrointestinal discomfort) (iv) EA + Herbal medicine/*n* = 8 (gastrointestinal discomfort, mild tension)
Kang 2015 [47]	(i) MA/*n* = 31 (ii) Estazolam/*n* = 33	(i) MA/47.50 ± 4.20 (ii) Estazolam/49.20 ± 3.90	(i) MA/15.90 ± 6.70 m (ii) Estazolam/16.60 ± 6.30 m	CCMD-3	(i) Heart and gallbladder *Qi* deficiency	(i) 40 min/day, 6 days/week for 4 weeks	EX, EX-HN1, GB13, GB15, GV16, GV20, GV24, scalp acupoint (1 *Cun* up to GB15)	(i) Estazolam 1 mg/day, 7 days/week for 4 weeks	(i) PSQI (ii) KI	(i) Compared with Estazolam *p* < 0.05 (ii) Compared with Estazolam *p* < 0.01	No follow-up	(i) MA/*n* = 0 (ii) Estazolam/*n* = 1 (mild nausea)
Lai 2016 [48]	(i) MA/*n* = 34 (ii) Eszopiclone/*n* = 33	(i) MA/51.28 ± 4.19 (ii) Eszopiclone/51.47 ± 4.03	(i) MA/8.33 ± 3.85 m (ii) Eszopiclone/9.08 ± 3.83 m	CCMD-3, CDTE-TCM	(i) Incoordination between heart and kidney	(i) 30 min/day, 6 days/week for 3 weeks (acupuncture at specific time)	BL62, KI6, LU7, SI3	(i) Eszopiclone 1 mg/day, 7 days/week for 3 weeks	(i) PSQI (ii) KI	(i) compared with Eszopiclone *p* > 0.05 (ii) compared with Eszopiclone *p* < 0.01	No follow-up	(i) MA/*n* = 2 (hematoma) (ii) Eszopiclone/*n* = 3 (one for dizziness; two for thirsty)
Li and Wang 2014 [49]	(i) MA/*n* = 120 (ii) Estazolam/*n* = 120	(i) MA/48.20 ± 0.00 (ii) Estazolam/47.80 ± 0.00	(i) MA/1.10 ± 0.20 y (ii) Estazolam/0.80 ± 0.20 y	CCMD-3	NR	(i) 30 min/day for 30 days	SP6, SP8, Shenguan	(i) Estazolam 2 mg/day for 30 days	(i) PSQI	(i) Compared with Estazolam *p* < 0.01	No follow-up	NR
Li et al. 2018 [50]	(i) MA/*n* = 60 (ii) Alprazolam/*n* = 62	(i) MA/51.00 ± 4.00 (ii) Alprazolam/50.00 ± 4.00	(i) MA/11.20 ± 5.20 m (ii) Alprazolam/10.20 ± 5.30 m	CDTE-TCM	NR	(i) 30–40 min/day, 5 days/week for 9 weeks	BL13, BL15, BL17, BL18, BL20, BL23, HT7	(i) Alprazolam 0.4–0.8 mg/day, 7 days/week for 9 weeks	(i) PSQI (ii) FSH (iii) E2 (iv) LH	(i) Compared with Alprazolam *p* < 0.05 (ii) Compared with Alprazolam *p* < 0.05 (iii) Compared with Alprazolam *p* < 0.05 (iv) Compared with Alprazolam *p* < 0.05	(i) Follow-up 30 days; NR for valid data	NR
Lu et al. 2014 [50]	(i) MA/*n* = 52 (ii) Estazolam/*n* = 52	(i) MA/49.70 ± 0.00 (ii) Estazolam/49.30 ± 0.00	(i) MA/3-7m (ii) Estazolam/2-6m	CCMD-3, ICD-10	NR	(i) 30 min/day for 30 days	(i) CV12, EX-HN1, GB20, GV20, HT7, LR3, LR14, SP6, SP15	(i) Estazolam 1 mg/day for 30 days	(i) PSQI	(i) MA compared with Estazolam *p* < 0.05	No follow-up	NR
Ma 2014 [46]	(i) EA/*n* = 45 (ii) Estazolam/*n* = 45	(i) EA/50.04 ± 2.67 (ii) Estazolam/50.42 ± 2.96	(i) EA/13.36 ± 7.47 m (ii) Estazolam/13.51 ± 7.76 m	CCMD-2	(i) Excessive Liver fire due to emotional suppression (ii) Disturbance of heart due to phlegm heat (iii) *Yin* deficiency leading to excessive fire (iv) Heart and spleen deficiency (v) Heart and gallbladder *Qi* deficiency	(i) 30 min/day, 3 days/week for 4 weeks (ii) Continuous wave, >50 Hz	PC6, SP6, Sishenzhen (1.5 *Cun* apart from GV20), Dingshenzhen (0.5 *Cun* up to EX-HN3, and 0.5 *Cun* up to GB14)	(i) Estazolam 1 mg/day, 7 days/week for 4 weeks	(i) PSQI (ii) HAMD	(i) Compared with Estazolam *p* < 0.01 (ii) Compared with Estazolam *p* < 0.01	No follow-up	No adverse events
Qin 2018 [51]	(i) MA/*n* = 34 (ii) Estazolam/*n* = 33	(i) MA/51.97 ± 2.27 (ii) Estazolam/50.85 ± 2.77	(i) MA/18.44 ± 7.55 m (ii) Estazolam/20.58 ± 9.25 m	CCMD-3, ICD-10, CDTE-TCM	(i) Deficiency of kidney and hyperactivity of liver	(i) 30 min/day, 5 days/week for 4 weeks	BL17, BL18, BL23, EX, EX-HN1, GV20, KI3, LR3	(i) Estazolam 1–2 mg/day, 7 days/week for 4 weeks	(i) PSQI (ii) HAMA (iii) light-sleep (%) (iv) deep-sleep (%) (v) REM (%) (iii)-(v) are recorded by MSMSMS	(i) Compared with Alprazolam *p* > 0.05 (ii) Compared with Estazolam *p* < 0.05 (iii) Compared with Estazolam *p* < 0.05 (iv) Compared with Estazolam *p* < 0.05 (v) Compared with Estazolam *p* < 0.05	No follow-up	(i) MA/*n* = 3 (hematoma) (ii) Estazolam/*n* = 7 (two for dizziness; two for daytime sleepiness; three for fatigue)
Yang et al. 2017 [52]	(i) MA/*n* = 81 (ii) Estazolam/*n* = 81	(i) MA/48.17 ± 4.12 (ii) Estazolam/49.45 ± 3.98	(i) MA/7.13 ± 1.96 m (ii) Estazolam/7.53 ± 2.11 m	CCMD-2	(i) Liver and kidney *Yin* deficiency	(i) 30 min/day, 15 days/month (one treatment every other day) for 3 months	CV12, HT7, KI3, PC6, ST36, ST40, four scalp acupoints (middle 1/3 of frontal apical band, posterior 1/3 of frontal apical band, anterior 1/3 of skull base band, middle 1/3 of skull base band)	(i) Estazolam 1 mg/day, 10 days/month for 3 months	(i) PSQI (ii) FSH (iii) E2 (iv) LH	(i) Compared with Estazolam *p* < 0.05 (ii) Compared with Estazolam *p* < 0.05 (iii) Compared with Estazolam *p* < 0.05 (iv) Compared with Estazolam *p* < 0.05	No follow-up	NR
Zhang et al. 2017 [53]	(i) MA/*n* = 31 (ii) Estazolam/*n* = 30	(i) MA/50.45 ± 3.50 (ii) Estazolam/48.97 ± 2.88	(i) MA/20.38 ± 20.53 m (ii) Estazolam/20.36 ± 20.44 m	GDTICA, CDTE-TCM	(i) six syndromes with liver as the core	(i) 30 min/day, 5 days/week for 4 weeks	BL17, BL18, EX, EX-HN1, GV20, LR3	(i) Estazolam 1 mg/day, 7 days/week for 4 weeks	(i) PSQI (ii) KI (iii) HAMA (iv) HAMD	(i) Compared with Estazolam *p* < 0.01 (ii) Compared with Estazolam *p* < 0.05 (iii) Compared with Estazolam *p* < 0.05 (iv) Compared with Estazolam *p* < 0.01	No follow-up	(i) MA/*n* = 1 (hematoma) (ii) Estazolam/*n* = 1 (two for dizziness, fatigue, and daytime sleepiness; two for memory loss)
Gao and Niu 2014 [54]	(i) MA + Estazolam/*n* = 32 (ii) Estazolam/*n* = 32	(i) MA + Estazolam/49.13 ± 2.47 (ii) Estazolam/49.50 ± 2.51	(i) MA + Estazolam/6.00 ± 3.12 m (ii) Estazolam/5.88 ± 2.70 m	CCMD-3	NR	(i) 20 min/day, 6 days/week for 4 weeks	EX-B2	(i) Estazolam 2 mg/day for 4 weeks	(i) PSQI	(i) Compared with Estazolam *p* < 0.05	No follow-up	NR
Ma 2016 [55]	(i) MA + Estazolam/*n* = 35 (ii) Estazolam/*n* = 35	(i) MA + Estazolam/49.80 ± 3.22 (ii) Estazolam/50.34 ± 2.99	(i) MA + Estazolam/10.74 ± 6.95 m (ii) Estazolam/10.91 ± 7.19 m	CCMD-3	NR	(i) 7 days/week for 4 weeks (NR for needle retention time)	EX, HT7, KI3, KI7, KI10, LR3, SP6, SP10, ST36	(i) Estazolam 2 mg/day, 7 days/week for 4 weeks	(i) PSQI (ii) FSH (iii) E2	(i) Compared with Estazolam *p* < 0.01 (ii) Compared with Estazolam *p* < 0.05 (iii) Compared with Estazolam *p* < 0.05	No follow-up	NR
Zhu et al. 2016 [56]	(i) MA + Estazolam/*n* = 37 (ii) Estazolam/*n* = 37	(i) MA + Estazolam/49.86 ± 3.15 (ii) Estazolam/49.27 ± 3.58	(i) MA + Estazolam/2.99 ± 4.24 m (ii) Estazolam/2.97 ± 3.42 m	CCMD-3	Heart and spleen deficiency	(i) 20 min/day, 5 days/week for 4 weeks (acupuncture at 15 : 00 P.M.-17 : 00 P.M.)	CV12, EX, EX-HN1, GV20, GV24, HT7, KI3, LR3, SP9, ST25	(i) Estazolam 1 mg/day, 5 days/week for 4 weeks	(i) PSQI	(i) Compared with Estazolam *p* > 0.05	No follow-up	NR

NR, no report; MA, manual acupuncture; EA, electroacupuncture; DSM-IV, Diagnostic and Statistical Manual of Mental Disorders (Fourth Edition); CCMD-2, Chinese Classification of Mental Disorders (Second Edition); CCMD-3, Chinese Classification of Mental Disorders (Third Edition); ICD-10, International Classification of Diseases (10th edition); GDTICA, Guidelines for Diagnosis and Treatment of Insomnia in Chinese Adults (2012 Edition); CDTE-TCM, Criteria of Diagnosis and Therapeutic Effect of Diseases and Syndromes in TCM; AIS, Athens Insomnia Scale; PSQI, Pittsburgh Sleep Quality Index; KI, Kupperman index; MENQOL, Menopause-Specific Quality of Life; HAMA, Hamilton Anxiety Scale; HAMD, Hamilton Depression Scale; WHOQOL-BREF, World Health Organization's quality of life scale-brief form questionnaire; MSMSMS, micromovement sensitive mattress sleep monitoring system; REM, Rapid eye movement sleep; FSH, follicle stimulating hormone; LH, luteinizing hormone; E2, estradiol; Progynova, Progynova (estradiol valerate tablets); BL13, Feishu; BL15, Xinshu; BL17, Geshu; BL18, Ganshu; BL20, Pishu; BL23, Shenshu; BL62, Shenmai; CV4, Guanyuan; CV12, Zhongwan; EX, Anmian; EX-B2, Jiaji; EX-CA1, Zigong; EX-HN1, Sishencong; EX-HN3, Yintang; GB13, Benshen; GB14,Yangbai; GB15, Toulinqi; GB20, Fengchi; GV14, Dazhui; GV16, Fengfu; GV20, Baihui; GV24, Shenting; HT7, Shenmen; KI3, Taixi; KI6, Zhaohai; KI7, Fuliu; KI10, Yingu; LR3, Taichong; LR14, Qimen; LU7, Lieque; PC6, Neiguan; SI3, Houxi; SP6, Sanyinjiao; SP8, Diji; SP9, Yinlingquan; SP10, Xuehai; SP15, Daheng; ST25, Tianshu; ST36, Zusanli; ST40, Fenglong; Shenguan, Tianhuangfuxue; six syndromes with liver as the core (liver stagnation (stasis); excessive liver fire due to emotional suppression; disturbance of liver *Yang*; deficiency of kidney and hyperactivity of liver; liver depression invading the stomach; liver depression invading the heart).

**Table 2:** Trends of major outcomes for sleep and perimenopausal symptoms in Acupuncture (or acupuncture + hypnotic) and comparison with controls in each study.

Author, year	Comparison		Outcome measures for sleep	Outcome measures for perimenopausal symptoms and sex hormone			
			PSQI	KI	FSH	E2	LH
Ma 2017 [42]	vs same group at different time-point	Post- vs pretreatment	↓	↓	(-)	↑	—
		3-month follow-up vs pretreatment	↓	↓	(-)	↑	—
		3-month follow-up vs posttreatment	(-)	(-)	(-)	(-)	—
	Acup vs HRT at same time-point	Posttreatment	<	(-)	(-)	<	—
		3-month follow-up	<	(-)	(-)	(-)	—
Chen et al. 2013 [43]	vs same group at different time-point	Post- vs pretreatment	↓ (use AIS instead of PSQI)	—	—	—	—
	Acup vs hypnotic at same time-point	Posttreatment	< (use AIS instead of PSQI)	—	—	—	—
Du et al. 2017 [44]	vs same group at different time-point	Post- vs pretreatment	↓	↓	↓	↑	—
	Acup vs hypnotic at same time-point	Posttreatment	<	<	<	>	—
Kang 2015 [46]	vs same group at different time-point	Post- vs pretreatment	↓	↓	—	—	—
	Acup vs hypnotic at same time-point	Posttreatment	<	<	—	—	—
Lai 2016 [47]	vs same group at different time-point	Post- vs pretreatment	↓	↓	—	—	—
	Acup vs hypnotic at same time-point	Posttreatment	(-)	<	—	—	—
Li 2014 [48]	vs same group at different time-point	Post- vs pretreatment	↓	—	—	—	—
	Acup vs hypnotic at same time-point	Posttreatment	<	—	—	—	—
Li et al. 2018 [49]	vs same group at different time-point	Post- vs pretreatment	↓	—	↓	↑	↓
		Follow-up vs pretreatment	no data	—	no data	no data	no data
	Acup vs hypnotic at same time-point	Posttreatment	<	—	<	>	<
Lu et al. 2014 [50]	vs same group at different time-point	Post- vs pretreatment	↓	—	—	—	—
	Acup vs hypnotic at same time-point	Posttreatment	<	—	—	—	—
Ma 2014 [45]	vs same group at different time-point	Post- vs pretreatment	↓	—	—	—	—
	Acup vs hypnotic at same time-point	Posttreatment	<	—	—	—	—
Qin 2018 [51]	vs same group at different time-point	Post- vs pretreatment	↓	—	—	—	—
	Acup vs hypnotic at same time-point	Posttreatment	(-)	—	—	—	—
Yang et al. 2017 [52]	vs same group at different time-point	Post- vs pretreatment	↓	—	↓	↑	↓
	Acup vs hypnotic at same time-point	Posttreatment	<	—	>	<	<
Zhang et al. 2017 [53]	vs same group at different time-point	Post- vs pretreatment	↓	↓	—	—	—
	Acup vs hypnotic at same time-point	Posttreatment	<	<	—	—	—
Gao et al. 2014 [54]	vs same group at different time-point	Post- vs pretreatment	↓	—	—	—	—
	Acup + hypnotic vs hypnotic at same time-point	Posttreatment	<	—	—	—	—
Ma 2016 [55]	vs same group at different time-point	Post- vs pretreatment	↓	—	↓	↑	—
	Acup + hypnotic vs hypnotic at same time-point	Posttreatment	<	—	<	>	—
Zhu et al. 2016 [56]	vs same group at different time-point	Post- vs pretreatment	↓	—	—	—	—
	Acup + hypnotic vs hypnotic at same time-point	Posttreatment	(-)	—	—	—	—

↑, statistically increase; ↓, statistically decrease; >, statistically higher/longer/more; <, statistically lower/shorter/less; (-), no statistical difference/no statistical changes; Acup, acupuncture; PSQI, Pittsburgh Sleep Quality Index; KI, Kupperman index; FSH, follicle stimulating hormone; LH, luteinizing hormone; E2, estradiol.

## Data Availability

This research is a systematic review, and all data were sourced from published articles.

## Supplementary Materials

Supplementary Materials Ten files are included in this paper as supplementary materials to support our research methods, results, and conclusions: (1) search strategy; (2) valid outcome measures at different time-point in each RCT; (3) risk of bias graph; (4) methodological quality assessment of 15 included RCTs; (5) details of acupuncture procedure based on revised STRICTA; (6) qualitative and quantitative analysis in 15 included RCTs; (7) acupuncture vs hypnotics in sleep scales; (8) criteria of effectiveness rate reported in the included studies; (9) subgroup analysis; (10) figures of meta-regression.
