# Supplementary material for: Comparative Utility of Acupuncture and Western Medication in the Management of Perimenopausal Insomnia: A Systematic Review and Meta-Analysis
Source: Evid Based Complement Alternat Med. 2021 Apr 26;2021:5566742. doi: 10.1155/2021/5566742 (PMC8093060; doi:10.1155/2021/5566742)

Supplementary Materials

Appendix 1. Search strategy

The following English search strategy was used for MEDLINE via PubMed, while it was also suitable for other English and Chinese electronic databases:

Medline search strategy for English database

Number	Search terms
1	((randomized controlled trial[Publication Type]) OR (controlled clinical trial[Publication Type]) OR (randomized[Title/Abstract]) OR (placebo[Title/Abstract]) OR (randomly[Title/Abstract]) OR (trial[Title]) OR ("Clinical Trials as Topic"[Mesh:noexp])) NOT ((animals[MeSH Terms]) NOT humans[MeSH Terms])
2	insomnia[MeSH Terms]
3	insomnia
4	Sleepless
5	sleep disorder
6	Sleep initiation dysfunction
7	Sleep maintain dysfunction
8	#2 or #3 or #4 or #5 or #6 or #7
9	acupuncture[MeSH Terms]
10	acupuncture
11	electropuncture
12	manual acupuncture
13	auricular acupuncture
14	acupuncture point
15	acupoint
16	#9 or #10 or #11 or #12 or #13 or #14 or #15
17	menopause
18	climacteric
19	climacterium
20	# 17 or #18 or #19
21	("1966/01/01"[Date - Publication] : "2020/03/31"[Date - Publication])
22	#1 and #8 and #16 and #20 and #21

PubMed Advanced Search Builder

PubMed.gov

User Guide

Add terms to the query box

Date - Publication | YYYY/MM/DD to Present | ADD

Query box

Enter / edit your search query here

Add to History

History and Search Details

Download Delete

Search	Actions	Details	Query	Results	Time
#22	•••	>	Search: **#1 and #8 and #16 and #20 and #21**	28	03:43:38
#21	•••	>	Search: **("1966/01/01"[Date - Publication] : "2020/03/31"[Date - Publication])**	28,402,440	03:42:43
#20	•••	>	Search: **# 17 or #18 or #19**	75,903	03:40:33
#19	•••	>	Search: **climacterium**	261	03:39:28
#18	•••	>	Search: **climacteric**	75,868	03:39:12
#17	•••	>	Search: **menopause**	85,553	03:38:42
#16	•••	>	Search: **#9 or #10 or #11 or #12 or #13 or #14 or #15**	34,730	03:33:40
#15	•••	>	Search: **acupoint**	11,656	03:33:29
#14	•••	>	Search: **acupuncture point**	10,506	03:33:20
#13	•••	>	Search: **auricular acupuncture**	1,448	03:33:11
#12	•••	>	Search: **manual acupuncture**	1,161	03:33:02
#11	•••	>	Search: **electropuncture**	71	03:32:50
#10	•••	>	Search: **acupuncture**	34,318	03:32:40
#9	•••	>	Search: **acupuncture[MeSH Terms]**	25,878	03:32:31
#8	•••	>	Search: **#2 or #3 or #4 or #5 or #6 or #7**	117,594	03:32:21
#7	•••	>	Search: **Sleep maintain dysfunction**	5,332	03:32:09
#6	•••	>	Search: **Sleep initiation dysfunction**	14,097	03:31:56
#5	•••	>	Search: **sleep disorder**	106,499	03:31:44
#4	•••	>	Search: **Sleepless**	14,703	03:31:34
#3	•••	>	Search: **insomnia**	27,099	03:31:22
#2	•••	>	Search: **insomnia[MeSH Terms]**	13,828	03:30:52
#1	•••	>	Search: **((randomized controlled trial[Publication Type]) OR (controlled clinical trial[Publication Type]) OR (randomized[Title/Abstract]) OR (placebo[Title/Abstract]) OR (randomly[Title/Abstract]) OR (trial[Title]) OR ("Clinical Trials as Topic"[Mesh:noexp])) NOT ((animals[MeSH Terms]) NOT humans[MeSH Terms])**	1,244,686	03:30:34

Showing 1 to 22 of 22 entries

Appendix 2. Valid outcome measures at different timepoint in each study

Author, year	Type of outcome measures	Pre-treatment	Post-treatment	Follow-up
Ma 2017 [42]	Sleep	PSQI	PSQI	PSQI (3 months)
	Perimenopausal symptoms	KI,MENQOL,FSH,E2	KI,MENQOL,FSH,E2	KI,MENQOL,FSH,E2 (3 months)
Chen et al 2013 [43]	Sleep	AIS	AIS	/
Du et al 2017 [44]	Sleep	PSQI	PSQI	/
	Perimenopausal symptoms	KI,FSH,E2	KI,FSH,E2	/
	Quality of life	WHOQOL-BREF	WHOQOL	/
Kang 2015 [46]	Sleep	PSQI	PSQI	/
	Perimenopausal symptoms	KI	KI	/
Lai 2016 [47]	Sleep	PSQI	PSQI	/
	Perimenopausal symptoms	KI	KI	/
Li 2014 [48]	Sleep	PSQI	PSQI	/
Li et al 2018 [49]	Sleep	PSQI	PSQI	No report of data (30 days)
	Perimenopausal symptoms	FSH,E2,LH	FSH,E2,LH	No report of data (30 days)
Lu et al 2014 [50]	Sleep	PSQI	PSQI	/
Ma 2014 [45]	Sleep	PSQI	PSQI	/
	Emotional symptoms	HAMD	HAMD	/
Qin 2018 [51]	Sleep	PSQI, light-sleep%, deep-sleep%, REM%	PSQI, light-sleep%, deep-sleep%, REM%	/
	Emotional symptoms	HAMA	HAMA	/
Yang et al 2017 [52]	Sleep	PSQI	PSQI	/
	Perimenopausal symptoms	FSH,E2,LH	FSH,E2,LH	/
Zhang et al 2017 [53]	Sleep	PSQI	PSQI	/
	Perimenopausal symptoms	KI	KI	/
	Emotional symptoms	HAMD,HAMA	HAMD,HAMA	/
Gao et al 2014 [54]	Sleep	PSQI	PSQI	/
Ma 2016 [55]	Sleep	PSQI	PSQI	/
	Perimenopausal symptoms	FSH,E2	FSH,E2	/
Zhu et al 2016 [56]	Sleep	PSQI	PSQI	/

Abbreviations: AIS, Athens Insomnia Scale; PSQI, Pittsburgh sleep quality index; KI, Kupperman index; HAMA, Hamilton Anxiety Scale; HAMD, Hamilton Depression Scale; WHOQOL-BREF, World Health Organization’s quality of life scale-brief form questionnaire; REM, Rapid eye movement sleep; FSH, follicle stimulating hormone; LH, luteinizing hormone; E2, estradiol

Appendix 3. Risk of bias graph

	Random sequence generation (selection bias)	Allocation concealment (selection bias)	Blinding of participants and personnel (performance bias)	Blinding of outcome assessment (detection bias)	Incomplete outcome data (attrition bias)	Selective reporting (reporting bias)	Other bias
Chen et al 2013	?	?	−	?	+	?	+
Du et al 2017	+	?	−	?	+	?	+
Gao et al 2014	?	?	−	?	+	?	+
Kang 2015	+	?	−	?	+	?	+
Lai 2016	+	+	−	+	+	?	+
Li 2014	?	?	−	?	+	?	+
Li et al 2018	+	?	−	?	+	−	+
Lu et al 2014	+	?	−	?	+	?	+
Ma 2014	+	+	−	?	−	?	+
Ma 2016	+	?	−	?	+	?	+
Ma et al 2017	+	?	−	?	+	?	+
Qin 2018	+	+	−	+	+	+	+
Yang et al 2017	?	?	−	?	+	?	+
Zhang et al 2017	+	?	−	?	+	?	+
Zhu et al 2016	+	?	−	?	+	?	+

Notes: other bias are assessed based on baseline balance, and source of funding or conflict of interest

Appendix 4. Methodological quality assessment of 15 included RCTs

Author, year	Random sequence generation	Allocation concealment	Blinding of participants	Blinding of personnel	Blinding of outcome assessment	Incomplete outcome data	Selective outcome reporting	Other bias (baseline balance)	Other bias (funding or conflict of interest)
Ma 2017 [42]	L	U	H	H	U	L	U	L	L
Chen et al 2013 [43]	U	U	H	H	U	L	U	L	U
Du et al 2017 [44]	L	U	H	H	U	L	U	L	U
Kang 2015 [46]	L	U	H	H	U	L	U	L	U
Lai 2016 [47]	L	L	H	H	L	L	U	L	U
Li 2014 [48]	U	U	H	H	U	L	U	L	U
Li et al 2018 [49]	L	U	H	H	U	L	H	L	U
Lu et al 2014 [50]	L	U	H	H	U	L	U	L	U
Ma 2014 [45]	L	L	H	H	U	H	U	L	U
Qin 2018 [51]	L	L	H	H	L	L	L	L	L
Yang et al 2017 [52]	U	U	H	H	U	L	U	L	L
Zhang et al 2017 [53]	L	U	H	H	U	L	U	L	L
Gao et al 2014 [54]	U	U	H	H	U	L	U	L	U
Ma 2016 [55]	L	U	H	H	U	L	U	L	U
Zhu et al 2016 [56]	L	U	H	H	U	L	U	L	L

Abbreviations: L, Low risk; U, Unclear risk; H, High risk.

Appendix 5. Details of acupuncture procedure based on revised STRICTA (2010 Version)

Item of STRICTA	*Studies* eligible to the items	Total number and percentage [n (%)]	Details of acupuncture procedure in each trial														
			Ma 2017 [42]	Chen et al 2013 [43]	Du et al 2017 [44]	Kang 2015 [46]	Lai 2016 [47]	Li 2014 [48]	Li et al 2018 [49]	Lu et al 2014 [50]	Ma 2014 [45]	Qin 2018 [51]	Yang et al 2017 [52]	Zhang et al 2017 [53]	Gao et al 2014 [54]	Ma 2016 [55]	Zhu et al 2016 [56]
1. Acupuncture rationale																	
(1a) Style of acupuncture	[42][43][44][45][46][47][48][49][50][51][52][53][54][55][56]	15 (100)	Chinese Acup	Chinese Acup	Chinese Acup	Chinese Acup	Chinese Acup	Chinese Acup	Chinese Acup	Chinese Acup	Chinese Acup	Chinese Acup	Chinese Acup	Chinese Acup	Chinese Acup	Chinese Acup	Chinese Acup
(1b) Rationale for treatment	[42][43][44][45][46][47][48][49][50][51][52][53][54][55][56]	15 (100)	TCM theory	TCM theory	TCM theory	TCM theory	TCM theory	TCM theory	TCM theory	TCM theory	TCM theory	TCM theory	TCM theory	TCM theory	TCM theory	TCM theory	TCM theory
(1c) Extent to which treatment was varied	/	0 (0)	NR	NR	NR	NR	NR	NR	NR	NR	NR	NR	NR	NR	NR	NR	NR
2. Needling details																	
(2a) Number of needles inserted	[42][43][44][45][46][47][48][49][50][51][52][53]	15 (100)	Reported	Reported	Reported	Reported	Reported	Reported	Reported	Reported	Reported	Reported	Reported	Reported	Reported	Reported	Reported

	[54][55][56]																
(2b) points used	[42][43][44][45] [46][47][48][49] [50][51][52][53] [54][55][56]	15 (100)	CV4; EX-CA1; EX-HN3; HT7; SP6; ST25	EX, GV20, HT7, KI3, KI7, KI10, LR3, PC6, SP6, SP9, SP10	PC6, SP6, Sishenzhen (1.5 *Cun* apart from GV20), Dingshenzhen (0.5 *Cun* up to EX-HN3, and 0.5 *Cun* up to GB14)	EX, EX-HN1, GB13, GB15, GV16, GV20, GV24, scalp acupoint (1 *Cun* up to GB15)	BL62, KI6, LU7, SI3	SP6, SP8, Shenguan	BL13, BL15, BL17, BL18, BL20, BL23, HT7	CV12, EX-HN1, GB20, GV20, HT7, LR3, LR14, SP6, SP15	PC6, SP6, Sishenzhen (1.5 *Cun* apart from GV20), Dingshenzhen (0.5 *Cun* up to EX-HN3, and 0.5 *Cun* up to GB14)	BL17, BL18, BL23, EX, EX-HN1, GV20, KI3, LR3	CV12, HT7, KI3, PC6, ST36, ST40, four scalp acupoints (middle 1/3 of frontal apical band, posterior 1/3 of frontal apical band, anterior 1/3 of skull base band, middle 1/3 of skull base band)	BL17, BL18, EX, EX-HN1, GV20, LR3	EX-B2	EX, HT7, KI3, KI7, KI10, LR3, SP6, SP10, ST36	CV12, EX, EX-HN1, GV20, GV24, HT7, KI3, LR3, SP9, ST25

(2c) Depths of insertion	[42][43][44] [45][47][48][49] [50][51][52][54]	11 (73.33)	0.5 *Cun*	0.5-1 *cun*	0.5-1 *cun*	NR	0.2-0.8 *cun*	1-1.5 *cun*	10-25 mm	2-20 mm	0.5-1 *cun*	0.5-1.2 *cun*	25-40 mm	NR	15-30 mm	NR	NR
(2d) Responses elicited	[42][43][44][45] [46][47][48][49] [50][51][52][53] [54][55][56]	15 (100)	*De-qi*	*De-qi*	*De-qi*	*De-qi*	*De-qi*	*De-qi*	*De-qi*	*De-qi*	*De-qi*	*De-qi*	*De-qi*	*De-qi*	*De-qi*	*De-qi*	*De-qi*
(2e) Needle stimulation	[42][43][44][45] [46][47][48][49] [50][51][52][53] [54][55][56]	15 (100)	EA	EA	EA	MA	MA	MA	MA	MA	EA	MA	MA	MA	MA	MA	MA
(2f) Needle retention time	[42][43][44][45] [46][47][48][49] [50][51][52][53] [54][56]	14 (93.33)	30 min	30 min	30 min	40 min	30 min	30 min	30-40 min	30 min	30 min	30 min	30 min	30 min	20 min	NR	20 min
(2g) Needle type	[42][43][45] [46][47][49] [50][51][54][56]	10 (66.67)	stainless steel (0.3*40 mm)	stainless steel (0.3*40 mm)	NR	stainless steel (0.3*40 mm)	stainless steel (0.3*25 mm for LU7; 0.3*40 mm for BL62, KI6, SI3)	NR	stainless steel (0.25*25 mm for HT7; 0.25*40 mm for BL13, BL15, BL17, BL18,	stainless steel (0.3*10 mm for GV16, HT7; 0.3*25 mm for CV12, EX-HN1, EX-HN3,	stainless steel (0.3*25 mm for Sishenzhen, Dingshenzhen; 0.3*50 mm for PC6, SP6)	stainless steel (0.25*25 mm for BL17, BL18, EX-HN1, GV20, KI3, LR3; 0.25*40 mm for	NR	NR	stainless steel (0.3*40 mm)	NR	stainless steel (0.3*25 mm for EX, EX-HN1, GV20, GV24, HT7, KI3, LR3; 0.3*40

									BL20, BL23)	GB20, GV14, LR3, LR14, SP6, SP15; 0.3*40 mm for GV20, GV24)		BL23, EX)					mm for CV12, SP9, ST25)
3. Treatment regimen																	
(3a) Number of treatment sessions	[42][43][44][45][46][47][48][49][50][51][52][53][54][55][56]	15 (100)	12 weeks	20 days	4 weeks	4 weeks	3 weeks	30 days	9 weeks	30 days	4 weeks	4 weeks	3 months	4 weeks	4 weeks	4 weeks	4 weeks
(3b) Frequency and duration of treatment sessions	[42][43][44][45][46][47][48][49][50][51][52][53][54][55][56]	15 (100)	30 min/day, 3 days/week for 12 weeks	30 min/day	30 min/day, 6 days/wee k	40 min/day, 6 days/wee k	30 min/day, 6 days/week	30 min/day	30-40 min/day, 5 days/wee k	30 min/day	30 min/day, 3 days/wee k	30 min/day, 5 days/wee k	30 min/day, 15 days/mo nth (one treatmen t every other day)	30 min/day, 5 days/wee k	20 min/day, 6 days/wee k	7 days/wee k	20 min/day, 5 days/wee k
4. Other																	

components of treatment																	
(4a) Details of other interventions administered to the acupuncture group	[54][55][56]	3 (20)	NR	NR	NR	NR	NR	NR	NR	NR	NR	NR	NR	NR	Estazolam	Estazolam	Estazolam
(4b) Setting and context of treatment	/	0 (0)	NR	NR	NR	NR	NR	NR	NR	NR	NR	NR	NR	NR	NR	NR	NR
5. Practitioner background																	
Description of acupuncturist	/	0 (0)	NR	NR	NR	NR	NR	NR	NR	NR	NR	NR	NR	NR	NR	NR	NR
6. Control or comparator interventions																	
(6a) Rationale for the control or comparator	[42][43][44][45][46][47][48][49][50][51][52][53][54][55][56]	15 (100)	Progynova + medroxyprogestone acetate	Alprazolam	Estazolam	Estazolam	Eszopiclone	Estazolam	Alprazolam	Estazolam	Estazolam	Estazolam	Estazolam	Estazolam	Estazolam	Estazolam	Estazolam
(6b)Precise	[42][43][44][45]	15 (100)	Reported	Reported	Reported	Reported	Reported	Reported	Reported	Reported	Reported	Reported	Reported	Reported	Reported	Reported	Reported

description of the control or comparator	[46][47][48][49] [50][51][52][53] [54][55][56]		(details in **Table 1**)	(details in **Table 1**)	(details in **Table 1**)	(details in **Table 1**)	(details in **Table 1**)	(details in **Table 1**)	(details in **Table 1**)	(details in **Table 1**)	(details in **Table 1**)	(details in **Table 1**)	(details in **Table 1**)	(details in **Table 1**)	(details in **Table 1**)	(details in **Table 1**)	(details in **Table 1**)

Abbreviations: NR, no record; Acup, acupuncture; TCM, Traditional Chinese Medicine; MA, manual acupuncture; EA, electroacupuncture; BL13, Feishu; BL15, Xinshu; BL17, Geshu; BL18, Ganshu; BL20, Pishu; BL23, Shenshu; BL62, Shenmai; CV4, Guanyuan; CV12, Zhongwan; EX, Anmian; EX-B2, Jiaji; EX-CA1, Zigong; EX-HN1, Sishencong; EX-HN3, Yintang; GB13, Benshen; GB14,Yangbai; GB15, Toulinqi; GB20, Fengchi; GV14, Dazhui; GV16, Fengfu; GV20, Baihui; GV24, Shenting; HT7, Shenmen; KI3, Taixi; KI6, Zhaohai; KI7, Fuliu; KI10, Yingu; LR3, Taichong; LR14, Qimen; LU7, Lieque; PC6, Neiguan; SI3, Houxi; SP6, Sanyinjiao; SP8, Diji; SP9, Yinlingquan; SP10, Xuehai; SP15, Daheng; ST25, Tianshu; ST36, Zusanli; ST40, Fenglong; Shenguan, Tianhuangfuxue; *De-qi* (obtaining Qi) refers to acupuncture-evoked specific sensations such as soreness, numbness, heaviness, and distention at the site of needle placement, and these sensations may spread to other parts of the body

Appendix 6. Qualitative and quantitative analysis in 15 included studies

Interventions *VS.* controls	Number of studies	Qualitative analysis	Quantitative analysis				
			pooled effect sizes	subgroup analysis	sensitivity analysis	meta-regression analysis	Publication bias
acupuncture *VS.* HRT	1	All	/	/	/	/	PSQI: 14 studies (one out of 15 studies used AIS instead of PSQI)
acupuncture *VS.* hypnotics	11	All	(i) PSQI: 10 studies<br>(ii) KI: 4 studies<br>(iii) FSH: 3 studies<br>(iv) E2: 3 studies<br>(v) Effectiveness rate: 10 studies	PSQI	PSQI	PSQI	
acupuncture + hypnotics *VS.* hypnotics	3	All	PSQI: 3 studies	/	/	/	

Abbreviations: HRT, hormone replacement therapy; AIS, Athens Insomnia Scale; PSQI, Pittsburgh sleep quality index; KI, Kupperman index; FSH, follicle stimulating hormone; E2, estradiol

Appendix 7 Acupuncture *Vs.* Hypnotics in sleep scales (PSQI and AIS)

	Acupuncture			Hypnotic				Std. Mean Difference	Std. Mean Difference
Study or Subgroup	Mean	SD	Total	Mean	SD	Total	Weight	IV, Random, 95% CI	IV, Random, 95% CI
Chen et al 2013	4.43	1.62	38	6.65	2.16	32	8.8%	-1.16 [-1.67, -0.65]	
Du et al 2017	6.71	2.04	41	9.4	2.48	41	9.0%	-1.17 [-1.64, -0.70]	
Kang 2015	9.26	3.99	31	11.42	4.54	33	8.9%	-0.50 [-1.00, 0.00]	
Lai 2016	10.18	3.75	34	10.82	3.5	33	9.0%	-0.17 [-0.65, 0.31]	
Li 2014	10.76	1.27	120	13.3	2.46	120	9.8%	-1.29 [-1.57, -1.01]	
Li et al 2018	6.72	1.24	60	6.9	1.89	62	9.6%	-0.11 [-0.47, 0.24]	
Lu et al 2014	5.85	2.22	52	10.19	2.53	52	9.1%	-1.81 [-2.27, -1.35]	
Ma 2014	6.18	1.77	45	11.16	2.19	45	8.6%	-2.48 [-3.03, -1.92]	
Qin 2018	6.56	2.45	34	7.33	2.2	33	9.0%	-0.33 [-0.81, 0.16]	
Yang et al 2017	5.93	1.41	81	8.4	2.95	81	9.7%	-1.06 [-1.39, -0.73]	
Zhang et al 2017	7.26	1.61	31	9.87	1.81	30	8.5%	-1.51 [-2.08, -0.93]	
**Total (95% CI)**			**567**			**562**	**100.0%**	**-1.05 [-1.44, -0.65]**	

Heterogeneity: Tau² = 0.40; Chi² = 94.37, df = 10 (P < 0.00001); I² = 89%

Test for overall effect: Z = 5.14 (P < 0.00001)

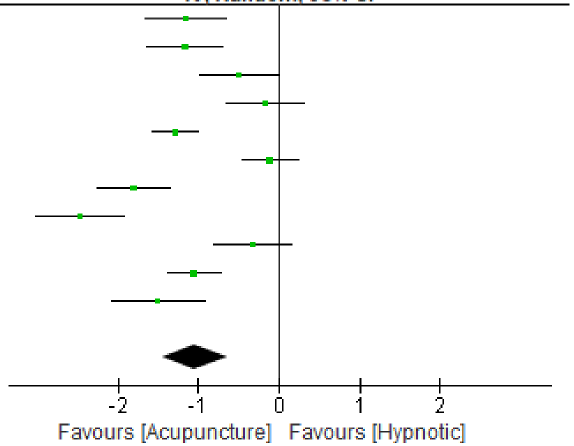

Appendix 8. Criteria of effectiveness rate reported in the included studies

Criteria	Details of guidelines	Involved studies	Frequency, n (%)
GCTNPCM	***Healed***: at the end of treatment, sleep pattern is back to normal and more than 6 hours per night	[42][43][46][47][49][52][54]	7 (46.67)
	Significant efficacious: sleep pattern is clearly improved and sleep time is increased by more than 3 hours		
	Efficacious: symptoms are relieved and sleep time is increased by less than 3 hours		
	Inefficacious: symptoms were not relieved		
Reduction rate of PSQI global scores	***Healed***: reduction rate of PSQI global scores ≥ 75%	[48][51][53][56]	4 (26.67)
	Significant efficacious: 75% > reduction rate of PSQI global scores ≥ 50%		
	Efficacious: 50% > reduction rate of PSQI global scores ≥ 25%		
	Inefficacious: reduction rate of PSQI global scores < 25%		
Sleep Efficiency Calculation published by WHO	**Healed:** all symptoms disappeared and sleep efficiency was higher than 75%	[45][50]	2 (13.33)
	Significant efficacious: symptoms alleviated and sleep efficiency was within 65-74%		
	Efficacious: symptoms slightly alleviated and sleep efficiency was within 55-64%		
	Inefficacious: symptoms were not relieved and sleep efficiency was lower than 55%		

Abbreviations: GCTNPCM, Guideline for Clinical Trials of New Patent Chinese Medicines; Reduction rate of PSQI global scores, reduction rate= [(PSQI global scores at pre-treatment - PSQI global scores at post-treatment)/(PSQI global scores at pre-treatment)] X 100%; sleep efficiency = (total sleep time/time in bed) X 100%

Appendix 9. Subgroup analysis of PSQI and KI (Acupuncture *Vs.* Hypnotic)

Basis for subgroup classification	All trials or subgroup title	No. of Studies	No. of participants	Statistical method	Effect size	*p*	I^2	Subgroup analysis results
PSQI	All trials	10	1059	Mean Difference (IV, Random, 95% CI)	-2.38 [-3.38, -1.37]	< 0.01	93	
Acupuncture stimulations	(i) MA	8	887	Mean Difference (IV< random, 95% CI)	-1.99 [-3.01, -0.98]	< 0.01	91	Chi^2 statistic 2.18, df = 1, *p* = 0.14
	(ii) EA	2	172	Mean Difference (IV< random, 95% CI)	-3.85 [-6.10, -1.61]	< 0.01	92	
Type of hypnotics	(i) Estazolam	8	870	Mean Difference (IV, Random, 95% CI)	-2.87 [-3.71, -2.03]	< 0.01	87	Chi^2 statistic 27.34, df = 2, *p* < 0.01
	(ii) Alprazolam	1	122	Mean Difference (IV< random, 95% CI)	-0.18 [-0.75, 0.39]	0.53	/	
	(iii) Eszopiclone	1	67	Mean Difference (IV, Random, 95% CI)	-0.64 [-2.38, 1.10]	0.47	/	
Acupuncture methods	(i) body acupuncture alone	6	661	Mean Difference (IV< random, 95% CI)	-1.88 [-3.17, -0.59]	< 0.01	94	Chi^2 statistic 1.72, df = 1, *p* = 0.19
	(ii) combination of body acupuncture and scalp acupuncture	4	398	Mean Difference (IV, Random, 95% CI)	-3.16 [-4.57, -1.75]	< 0.01	87	
KI	All trials	4	274	Mean Difference (IV, Random, 95% CI)	-5.95 [-10.68, -1.21]	< 0.01	96	
Acupuncture stimulations	(i) MA	3	192	Mean Difference (IV< random, 95% CI)	-6.24 [-12.78, 0.30]	0.06	97	Chi^2 statistic 0.12, df = 1, *p* = 0.73
	(ii) EA	1	82	Mean Difference (IV< random, 95% CI)	-5.05 [-7.09, -3.01]	< 0.01	/	

Type of hypnotics	(i) Estazolam	3	207	Mean Difference (IV, Random, 95% CI)	-3.88 [-6.12, -1.63]	< 0.01	71	Chi² statistic 30.43, df = 1, *p*< 0.01
	(ii) Eszopiclone	1	67	Mean Difference (IV, Random, 95% CI)	-11.61 [-13.19, -10.03]	< 0.01	/	
Acupuncture methods	(i) body acupuncture alone	2	128	Mean Difference (IV< random, 95% CI)	-6.80 [-16.22, 2.61]	0.16	99	Chi² statistic 0.13, df = 1, *p* = 0.72
	(ii) combination of body acupuncture and scalp acupuncture	2	146	Mean Difference (IV, Random, 95% CI)	-5.06 [-6.70, -3.41]	< 0.01	0	

Abbreviations: MA, manual acupuncture; EA, electroacupuncture

Appendix 10. Figures of meta-regression

Supplemental Figure 1. Univariate meta-regression based on study sample size as covariate

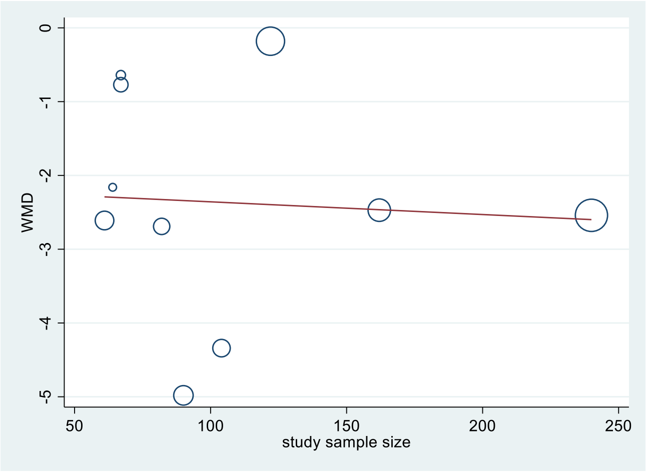

Supplemental Figure 2. Univariate meta-regression based on acupuncture stimulation as covariate

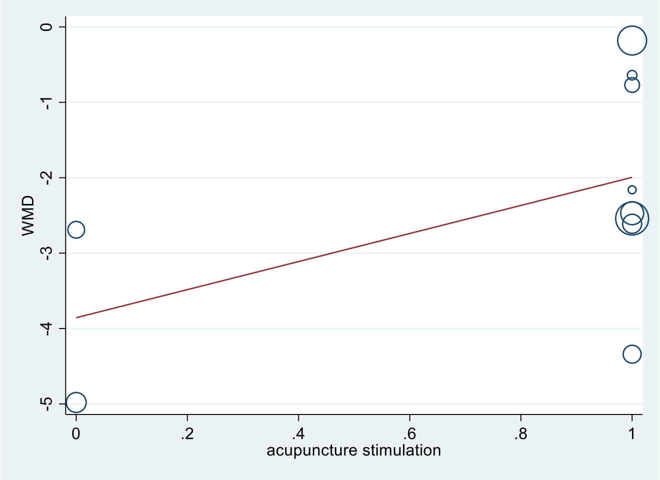

Supplemental Figure 3. Univariate meta-regression based on acupuncture methods as covariate

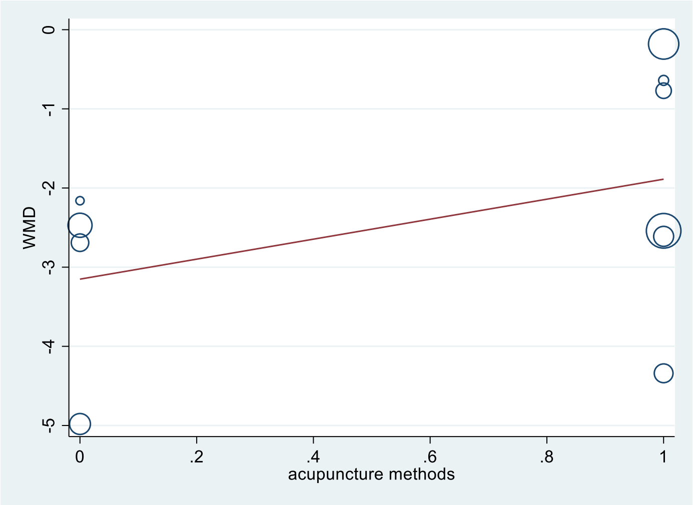

Supplement: Supplementary Materials — Ten files are included in this paper as supplementary materials to support our research methods, results, and conclusions: (1) search strategy; (2) valid outcome measures at different time-point in each RCT; (3) risk of bias graph; (4) methodological quality assessment of 15 included RCTs; (5) details of acupuncture procedure based on revised STRICTA; (6) qualitative and quantitative analysis in 15 included RCTs; (7) acupuncture vs hypnotics in sleep scales; (8) criteria of effectiveness rate reported in the included studies; (9) subgroup analysis; (10) figures of meta-regression. [file 5566742.f1.pdf]
